# Parents’ Beliefs about Medicines and Their Influence on Inhaled Corticosteroid Adherence in Children with Asthma

**DOI:** 10.3390/children11020167

**Published:** 2024-01-27

**Authors:** Jasna Petrić Duvnjak, Anita Ursic, Antonela Matana, Ivana Medvedec Mikic

**Affiliations:** 1 Pediatric Clinic “Pediatri”, 21000 Split, Croatia; jpetric@mefst.hr (J.P.D.); anita.ursic@mefst.hr (A.U.); 2 School of Medicine, University of Split, 21000 Split, Croatia; 3 Department of Health Studies, University of Split, 21000 Split, Croatia; antmatana@ozs.unist.hr; 4 Department of Maxillofacial Surgery, University Hospital of Split, 21000 Split, Croatia

**Keywords:** asthma, anti-asthmatic agents, adherence, inhalation drug administration, pediatrics

## Abstract

The most widespread chronic condition observed amid children globally is asthma. Only half of children with asthma adhere to their prescribed inhaled corticosteroids (ICS) therapy. Parents’ emotions and perspectives regarding asthma have an impact on inhalation corticosteroid adherence. The participants in this study were 148 parents of children with asthma, with the aim to redintegrate their beliefs about medicines in general and specifically of ICS and the impact on ICS adherence in children with asthma. Children were mostly male (66.9%), older than five years (58.8%), parents were female, mean age 38, employed, and with a history of consumption of some form of corticosteroids. Parents’ answers show that 50% of them disagreed with the statement that medicines are addictive, and 90% agree that medicine helps many to live better. A percentage of 77.7% of parents acknowledge that their child’s health relies on inhaled corticosteroids (ICS), and 86.5% of parents agree that these medications safeguard their child from worsening health. Most of the parents (93.2%) adhere to the guidelines and instructions of the doctor. In summary, parents who hold the belief that medicines are neither overused nor harmful tend to exhibit a higher adherence. Furthermore, those with elevated adherence levels express lower levels of concern regarding the use of inhaled corticosteroids (ICS) in their children’s asthma therapy.

## 1. Introduction

The most widespread chronic condition observed among children globally is asthma [1]. According to Global Burden of Disease (GBD) data in Europe, in the year 2019 approximately 5.5 million children under age 14 suffer from asthma. Prevalence was higher in Western European countries (6.38%), and lower in Eastern (4.78%). The prevalence of children’s asthma in Croatia was 4.66% [2]. 

Despite significant progress in understanding the pathophysiology of asthma in the past three decades, particularly its inflammatory characteristics and the existence of exceptionally potent medications like inhaled corticosteroids (ICS) to manage the condition, numerous children with asthma continue to struggle with attaining sufficient control over their symptoms [3]. Recent data from the Global Asthma Network (GAN) Report 2022, emphasizes a much higher frequency of utilization of inhaled or even oral short-acting β2-agonists than any other asthma medication in children aged 6–7 [4], indicating a prevalent reliance on relievers instead of recommended controller asthma medication.

Uncontrolled asthma was reported in 25.3% of children, opposite to 16% of adults [5]. Unfortunately, a common cause of unsuccessful asthma management was low adherence of ICS treatment leading to recurrent asthma attacks. As a result of asthma exacerbation children who suffer from asthma, compared to their peers, have a three times higher risk of school absence [6]. In addition to acute worsening, long-term day and night symptoms with activity restrictions greatly reduce the life quality of the parents and child [7,8].

It has been observed that only half of children with asthma adhere to their prescribed ICS therapy [9] due to still not fully clear reasons. Insufficient use of ICS treatment carries the potential risk of exacerbating asthma symptoms, even among individuals with only mild symptoms [10].

In the case that a child with uncontrolled asthma is not recognized as being nonadherent to their inhaled controller therapy, usually ICS, it can direct clinicians to incorrectly believe that the patient is unresponsive to the initial treatment. This misconception often leads to expensive diagnostic tests to understand why the child is not responding well to the treatment [11]. Similarly, it can also result in an unneeded increase in medication dosage and escalation to more costly controller therapies, sometimes even reaching the level of biological treatment [12].

Parents of children who have asthma withstand a crucial obligation to monitor symptoms and effectively carry out controller therapy [13]. The choice made by parents to adhere to the doctor’s advice for their children’s health is significantly influenced by their own emotions and perspectives regarding asthma and the recommended medication. They consider both the seriousness of the illness and the susceptibility of their children, while also recognizing the importance or additional benefits of the medications against the actual or potential risks or drawbacks associated with the advised medications, which may include potential side effects [14]. 

Adherence, as defined by the World Health Organization (WHO), explains the girth to which the patient’s or, in this case, parents’ behavior corresponds to the agreement about treatment with the physician [15,16] and is one of the prerequisites for successful treatment. Experts have put forward a multitude of potential factors, related to patient, medication, illness, healthcare system, logistical, financial, or sociocultural issues [17] estimated to be around 200 of them, that could potentially impact the level of adherence [18]. 

Nonadherence could be intentional (perception, concerns, and beliefs about medicine and disease), unintentional (misunderstanding instructions, forgetfulness), or medication-regime related (multiple devices, multiple times per day) [19].

How parents prioritize their concerns related to perceived requirements has been identified as a predictor of medication adherence. Various barriers that can be altered may directly stem from inadequate communication and relationships between parents and physicians. This often occurs during brief consultations where insufficient education is provided, leading to misunderstandings or negative beliefs among parents regarding necessary medications. Typically, there is little or no discussion about these concerns with healthcare providers [20]. Other barriers include worries about potential medication side effects [21] or the misconception that the truancy of symptoms implies the truancy of disease. As a result, some parents would rather give controller medication to their children only through symptomatic phases instead of implementing constant controller therapy [22].

The objectives of this research were to estimate the extent to which parents of children with asthma aged 2–10 adhere to the use of ICS and what influences adherence has on parents’ beliefs about medicines in general and specifically about ICS. At the moment, there is a scarce amount of research on this topic in this specific population.

## 2. Materials and Methods

### 2.1. Study Design 

From April 2023 to July 2023, a cross-sectional study in the form of a questionnaire was carried out in the city of Split, located in Croatia. In this study, we enrolled parents and children who were attending regular, prearranged appointments with pediatric pulmonologists for asthma-related visits. While waiting, parents completed the questionnaires electronically using tablets including: demographic data, beliefs about medicines general (BMQ-G), beliefs about medicines specific (BMQ-S), and medication adherence report scale (MARS) [23]. Information obtained from children’s medical records included details regarding their age, gender, age at which the disease began, allergic sensitization on inhalational or food allergens, IgE levels, presence of other parents or children’s medical conditions (allergic rhinitis, atopic dermatitis, food or medicine allergies), family history of asthma, previous hospitalizations or emergency visits because of asthma exacerbations, and alternative and complementary medicine use. The pediatric pulmonologist evaluated the degree of asthma symptom control during the previous four-week period, following the guidelines provided by GINA (Global Initiative for Asthma) that include activity limitation, daytime and nighttime symptoms, and use of reliever medication (short-acting beta agonist, SABA). Based on the GINA guidelines, children were divided into two groups because of different assessment of symptom control. This study involved the participation of parents or legal guardians of children from 2 to 10 years old who had previously been diagnosed with asthma by a pediatric pulmonologist based on the GINA guidelines. Additionally, these children had been prescribed daily inhaled corticosteroids (ICS) for a minimum of three months. The study did not include patients who had acute illnesses or acute asthma exacerbation, as well as children who were receiving treatment with ICS for reasons unrelated to asthma. Before involvement in the study, all individuals provided their informed consent. The study adhered to the guidelines outlined in the Declaration of Helsinki, and the Ethics Committee of the School of Medicine, University of Split (003-08/23-03/0015, 20 April 2023) approved the protocol. 

### 2.2. Demographic Information 

The gathered information about the population consisted of various aspects related to their parents. These aspects included their age, gender, overall health condition, educational background, employment situation, previous tobacco use, number of children, presence of asthma or any other form of allergy, and past usage of corticosteroids.

### 2.3. Beliefs about Medicines General Questionnaire

The initial version of the questionnaire was translated and confirmed as accurate or reliable. The Beliefs about Medicines General Questionnaire (BMQ-G) included 12 items with a five-point Likert scale (where 1 = strongly disagree, 2 = disagree, 3 = uncertain, 4 = agree and 5 = strongly agree). There are three groups of statements regarding overuse (3 items), harm (5 items), and benefit (4 items) of medicines in general. The Cronbach’s αs showed that all scale measures were internally coherent in the study sample (α = 0.600, α_Harm_ = 0.720, α_Benefit_ = 0.681, α_Overuse_ = 0.737). A mean score on the scale was calculated using the total of each item score divided by the number of items (e.g., mean score of BMQ-G-Overuse = (O1 + O2 + O3)/3). 

### 2.4. Beliefs about Medicines Specific Questionnaire

The original parent’s version of the Beliefs about Medicines Specific Questionnaire (BMQ-S) contained 11 statements with a five-point Lickert scale. This questionnaire was translated into the Croatian language and confirmed as accurate or reliable. It consists of two groups of statements about ICS. Five items assessed parents’ opinions about the necessity of ICS usage and the other six were about parents’ concerns about ICS’s long-term effects and side effects. Any number from 5 points (strongly agree) to 1 point (strongly disagree) was scored for each item. The Cronbach’s αs indicated that all scale measures were internally consistent in the study sample (α = 0.680, α_Necessity_ = 0.732, α_Concern_ = 0.728). At the midpoint, we divided subscales of necessity and concerns based on the authors’ instructions [23]. Scores above the midpoint, for the necessity subscale (>15) or concern subscale (>18) indicated strong beliefs (necessity or concern). The necessity-concern differential was distributed in three groups: greater concern than necessity score, greater necessity than concern score, and group of parents with equal necessity and concern score. 

### 2.5. Medication Adherence Report Scale

The Medication Adherence Report Scale (MARS) comprised six statements about the way that parents give prescribed ICS to their children. Parents had to choose from a five-point Lickert scale (always to never) about whether they forget or stop giving the dose, alter the dose, give less or miss out the dose of ICS, or even do not give it because the child refused it. Answers were scored for a final adherence score in the range of six to thirty, with a higher score representing a higher adherence to prescribed ICS. Parents’ version of the questionnaire was translated and validated (Cronbach α = 0.778).

### 2.6. Statistical Analysis

We used the Kolmogorov–Smirnov test to check for normality. The median presented continuous variables (interquartile range, IQR) or mean ± standard deviation, depending on the distribution of the data. Frequencies (percentages) were expressed with categorical variables. For non-normally distributed continuous variables, we used Mann–Whitney and Kruskal–Wallis tests. The Spearman rank correlation test was used to analyze correlations between non-normally distributed variables. Two-sided *p*-values less than 0.05 were considered statistically significant. The Statistical Package Software for Social Science, version 28 (SPSS Inc., Chicago, IL, USA) was used for statistical analysis.

## 3. Results

### 3.1. Descriptive Characteristics of Children

In total, 148 children with asthma were involved in this study; 99 (66.9%) of them were male. The median age was 5.65 (IQR: 5.52, 2 to 10 years old). Children were split into two groups: younger than five years 61 (41.2%) and older than five years, 87 (58.8%). Data about allergic sensitization, family history of atopy, pets, comorbidities (allergic rhinitis, atopic dermatitis, and food and drug allergies), admissions to the emergency room due to asthma, hospitalization, exacerbation of asthma 4 weeks before the visit, playing sports, and alternative treatment usage can be seen in Table 1. Asthma symptom control was estimated according to the GINA assessment and consisted of questions about the daytime and nighttime symptoms in past four weeks, limitation of activities and reliever (SABA) medication use. If the child did not experience any daytime symptoms more than a few minutes, activity limitation, night waking or coughing, or using SABA more than once a week for children 5 years and younger or more than twice a week for children 6 years old and older they had well-controlled level of asthma. Children who were using relievers before exercise were excluded from the assessment. 

### 3.2. Descriptive Characteristics of Parents

The parents or legal guardians average age was 38 (ranging from 26 to 67), and all of them were females. Their data can be seen in Table 2.

### 3.3. Results of the Beliefs about Medicines General Questionnaire

Among the 148 participants, 58.1% expressed strong disagreement or disagreement with the assertion that doctors excessively prescribe medications. As for the statement that doctors place too much trust in medicines, most parents were uncertain (42%). A similar situation is seen with the statement about doctors’ time spent with patients and its impact on prescribing medicine. Even 38.5% of parents are uncertain.

Parents’ answers about the harm of the medicines show that 50% of them disagree with the statement that medicines are addictive. Only 6.8% of parents think that medicines are poisons and 75% of them disagree or strongly disagree that medicines do more harm than good. Many participants (40.5%) were uncertain about the statement that people who take medicines should stop their treatment for a while now and again. The same situation is seen with the opinion about the statement that natural remedies are safer than medicines. A total of 47.3% of parents were uncertain. 

Regarding the benefits of medicines, 90% of participants agree or strongly agree that medicine helps many people to live better lives. That the benefits of medicines outweigh the risks 57% of parents agree, and 82% of parents agree that medicines help many people to live longer. The statement “In the future medicines will be developed to cure most diseases“ challenged 70% of respondents to answer agree or strongly agree (Table 3).

### 3.4. Results of the Beliefs about Medicines Specific Questionnaire

In total, 77.7% of parents concur that a child’s health is contingent upon the use of inhaled corticosteroids (ICS), while 51.3% agree that without ICS, their child would be at risk of significant illness.

A very high percentage (86.5%) of parents agree that these ICS protect their child from being sick. The highest percentage of parents (48%) were not certain whether their child’s health in the future will depend on ICS and one-third of parents disagreed or were uncertain about the statement that their child’s life would be harder without these medicines. 

The other part of the questionnaire about parent’s concerns shows that 54.8% of parents are worried about the fact that their child has to take ICS. A high percentage is worried about the long-term effects of ICS (65.5%). Contrarily, 77.1% of parents disagree with the statement that ICS causes their child unpleasant side effects, and 86% disagree with the statement that ICS disrupts their child’s life. That their child will become too dependent on ICS concerns 55.4% of parents and only 12.2% of tested parents find their child’s medicines (ICS) a mystery to them (Table 4). 

Among parents, 21.6% harbored significant worries regarding the use of inhaled corticosteroids (ICS), while a substantial 67.6% firmly believed in the essentiality of ICS for their child’s well-being. Necessity outweighed concerns for 47.3% of parents, while the opposite was true for 40.5%. A comparable necessity and concern score was observed in 12.2% of cases.

### 3.5. Results of the Medication Adherence Report Scale (MARS)

Answers to this questionnaire show that most of the tested parents rarely or never do the following: forget to give medicines to their children, change their doses, stop giving medicines to their children, skip one or more doses, use fewer medications than are prescribed, and do not give the medicine because the child refuses it (Table 5). 

Most of the parents adhere to the guidelines and instructions on the use of the medications. Based on the score of the MARS questionnaire, a total of 10 parents (6.8%) had less than 24 points and they were the low MARS adherent group. More than 24 points had 138 parents (93.2%) and they were the MARS adherent group. Fully adherent were 28.4% of parents (score of 30). 

### 3.6. Correlations between Demographic Variables and All Three Questionnaires

Comparing the demographic variables like age, education level, employment status, etc., and all three questionnaire results, we found that older parents had lower MARS scores (*p* = 0.036; r = −0.176), lower BMQ g Harm scores (*p* = 0.023; r = −0.186) and a higher BMQ-S Necessity score (*p* = 0.016; r = 0.198), suggesting that older parents have weaker adherence but think that medicines, in general, are not harmful and that ICS is necessary for their children. 

The level of education also had an impact on parents’ opinions about medicines in general. Parents who finished only high school were more likely to think that medicines are harmful, while those with master’s degrees or a PhD were more likely to consider medicines in general not harmful. As for employment status, unemployed parents found medicines in general harmful and were concerned about ICS usage more than employed parents.

Parents without a medical education had higher BMQ g Harm scores, that is, they think that medicines, in general, are harmful. Non-smoking parents had higher scores of BMQ g Benefits than smoking. And finally, parents who practice alternative medicines find medicines in general not harmful to their children.

### 3.7. Correlations between All Three Questionnaires

Correlating the answers from all three questionnaires, we found that MARS and BMQ g Overuse have a statistically significant negative correlation suggesting that parents with a higher adherence think that medicines in general are not overused (r = −0.235, *p* = 0.004). A borderline significance was observed for the MARS and BMQ Harm questionnaire (r = −0.156, *p* = 0.058). Correlating the results from the MARS and BMQ-S Concern questionnaire, one can conclude that parents with a higher adherence are less concerned about ICS usage (r = −0.214, *p* = 0.009).

BMQ g Harm and BMQ g Benefit’s results are in negative correlation, and it is statistically significant (r = −0.328, *p* < 0.001) meaning that parents who find medicines in general harm consider them less useful.

The opposite is found with the results of the correlation between BMQ g Harm and BMQ-S Concern which is positive (r = 0.365, *p* < 0.001). One possible explanation is that parents who perceive medicines in general as harmful may express heightened concerns regarding their child’s use of inhaled corticosteroids (ICS).

Answers to the BMQ g Benefit and the BMQ-S Necessity questionnaire have a statistically significant positive correlation (r = 0.170, *p* = 0.038) suggesting that parents who think that the benefits of the medicines in general are weak consider ICS less useful and necessary.

BMQ g Overuse and BMQ-S Concern results when put into correlation lead to the conclusion that parents who think that medicines in general are overused were more concerned about ICS treatment of their children (r = 0.409, *p* < 0.001).

## 4. Discussion

As per the existing literature, the first symptoms of asthma manifests in 80% of instances during the initial five years of a child’s life “[24]” and exhibits a higher prevalence in boys until the age of 10 “[25]”. In our study, this prevailed in boys older than five years, as this age group constitutes the most common demographic among asthma patients in our pediatric pulmonology practice. Factors that increase the likelihood of developing asthma in children [19] were assessed and other allergic diseases like allergic rhinitis, atopic dermatitis, or food/drug allergies were noted in almost three-quarters of the children of our participants. In this study, more than half of the children were found to be sensitized to common aeroallergens, comparable to data reported by Arbes et al. “[26]” which emphasized the crucial role that early onset allergic sensitization plays in predicting the development of persistent asthma [27]. The majority of the children in our respondents’ families had a positive family history of food allergies, atopic dermatitis, or allergic rhinitis. A significant 69.6% of all family members exhibited a history of asthma while only 14.9% of parents had a history of asthma. These findings underscore the importance of obtaining a comprehensive family history, as the likelihood of asthma development increases with the number of family members affected “[28]”. 

In this study, 99 percent of respondents were mothers, who were predominantly employed and high school educated. The reason for that could be the fact that mothers are primary health caregivers and play an important role in their children’s lives as published in a previous study [29].

There are few studies that investigate parents’ beliefs about medicines in general. In this study, only few of the participants agree that most medicines are poisons, suggesting that our participants do not have trust in medicines in general. Level of education and religion can have an impact on opinion about medications in general [30]. Still, these beliefs could be changed by healthcare intervention or advice [31]. According to the literature, individuals with lower educational levels tend to perceive medicines in general as more harmful, which is not the case with those with higher education levels [32]. The results of this study follow this; parents who finished only high school more often think that medicines in general are harmful while parents with master’s degrees or PhDs more often consider medicines in general not harmful. Parents with medical education also consider medicines in general less harmful, which is in line with Swedish authors who reported that medical professionals find medicines, in general, less harmful than patients [33]. Similar to the findings of Hong et al. [34], our research also indicates that unemployed parents tend to hold a stronger belief in the potential harm of medicines compared to employed individuals. This association may be attributed to the influence of unemployment on psychological distress, levels of optimism, and trust in institutions [35]. Parents’ age was also one of the factors that influenced their opinion about medicines in general in a way that older parents do not consider drugs in general harmful. Opposite to these results, Barakat et al. reported that a high corticophobia score in the general population was positively correlated with age and negatively with educational level “[36]”.

Parents who were using complementary and alternative medicine (CAM) as a part of their children’s therapy had lower BMQ general harm scores in our population, perhaps because CAM are also pharmacy products like vitamin D and C, multivitamins, probiotics, and immunoglucan, which are often given to children to strengthen immunity.

Based on the results of the BMQ-S questionnaire, 21.6% of our participants have strong concerns about using ICS for their children’s asthma therapy even though only 3.4% of them reported unpleasant side effects of ICS. Probably because of a strong, negative, image of corticosteroids in general. Similar results were reported in the study of Zedan et al. [37] with oral steroid-treated children, where parents were afraid of steroid side effects and were worried about non-specific side effects. Unemployed parents were more worried about ICS use. There is scarce evidence about the impact of parents’ employment on beliefs about medicines. There are only pieces of information about the significant impact of family income on concerns about ICS use [38]. The fact that parents were worried about the long-term effects of ICS on their children (65.5%) could be the reason why a high number of parents wanted to stop giving ICS to children as quickly as possible. The outcome stems from earlier research carried out on the identical population using the TOPICOP questionnaire. [39]. Opposite the above-mentioned results, a high percentage of parents believe they have good knowledge about ICS, the same as in the study of Klok et al. [29]. Just one-quarter of parents think their children will become dependent on ICS usage and only 4.8% of them reported that ICS disrupts a child’s life. Parents in general agree that their child’s health now depends on using ICS and that they are necessary to keep their children healthy. In a study published by Conn, 75% of parents shared this opinion. Contrarily, two studies by Koster et al. [40] (41.9%) and Kosse et al. [41] (42.8%) carried out on adolescents, respectively, reported significantly lower percentages. One can conclude that parents are more aware of the importance of ICS use in the therapy of asthma than adolescents are. 

Looking at the necessity/concern score from the BMQ-S questionnaire, it can be seen that in this research, the ratio was on the side of necessity. One of the conclusions could be that parents are aware that ICS is necessary medication for a therapy of their children’s asthma. The same opinion had the participants of the research conducted by Conn et al. [14] on parents of children aged 2–16 treated also with preventive medicine (ICS, a leukotriene inhibitor, etc.) but their score was much bigger.

That stronger beliefs about the harmfulness of medications were associated with poorer adherence was reported in a systematic review of studies conducted on adult patients with chronic diseases [42]. However, this correlation was observed in certain populations but not across all, suggesting that cultural factors may influence individuals’ beliefs about harm [42]. 

That parents who think that medicines in general are not overused have higher adherence showed the results of this study. Similar results were published in the study conducted on the adult German population with chronic lung diseases [43]. Conversely, three separate studies involving chronic adult patients with good health literacy and higher educational backgrounds, spanning from high school to college graduates, did not establish a correlation between medication adherence and general beliefs about medications [42]. A recent study has proved that effective communication between patients and physicians, especially interpersonal connections, significantly influences beliefs regarding medication overuse [34].

A study conducted in the Netherlands reported that low maternal education level was associated with uncontrolled asthma in children aged 8 most probably as a consequence of lower adherence [21]. Perhaps a lack of familiarity with medications, including general pharmaceutical knowledge and understanding of inhaled corticosteroids (ICS), might lead to diminished comprehension of medical guidance. This could result in a failure to grasp the potential complications that may arise from non-compliance with the prescribed instructions.

A study by Clifford et al. proved that 10 days after the beginning of chronic condition therapy significant differences in beliefs about medicine had arisen between adherent and non-adherent patients [18]. Patients who deliberately fail to follow their prescribed medication regimen were found to have a higher tendency to prioritize their worries about the medication over their perceived necessity for the treatment. In our study, parents who felt that drugs are generally harmful were also worried about the use of ICS in the therapy of their children and had lower adherence, so it could be that previous experience with other medicine treatments and medicine, in general, accelerated that response of intentional nonadherence. By identifying the key factors that impact these beliefs, it becomes possible to develop a patient-centered communication program that encourages positive medication beliefs.

Looking at the influence of parents’ age, older parents feel that ICS is necessary in their children’s therapy, the same as in the research of Conn et al. [14], and also consider drugs in general less harmful, but surprisingly, they had lower adherence. Perhaps older parents have more experience and think that asthma will resolve on its own at some point. This is supported by the fact that despite being classified as a long-term condition during childhood, the trajectory of asthma symptoms can differ significantly [24,44]. Nearly 80% of individuals diagnosed with asthma encounter symptoms within the initial six years of their life. However, it is noteworthy that among children who have asthma at the age of seven, approximately 67–75% will ultimately become free from symptoms upon reaching adulthood [45,46]. 

Different results about the connection of CAM and adherence have been published recently. Adams et al. found that strong parental beliefs are closely linked with increased risks of nonadherence to prescribed treatments and suboptimal asthma control [47]. However, the contrary longitudinal study by Chen et al. found no link between the use of CAM and adherence to asthma controller therapy in children [48]. Even though around 75% of our participants give their asthmatic children CAM, almost half of all parents were uncertain whether natural remedies are safer than medicines. The findings indicate that parents may adopt multiple health belief systems concurrently when managing their children’s asthma and the utilization of CAM does not inherently compete with traditional treatments for asthma. 

As mentioned before, adherence represents the extent to which parents of children with asthma adhere to the doctor’s instructions. As per the World Health Organization (WHO), prioritizing and improving adherence to guidelines and recommendations could potentially offer more significant benefits for the health and prosperity of the population when compared to focusing solely on specific medical interventions [16]. These discoveries possess substantial importance within clinical environments as they assist in recognizing parents who could be prone to neglecting mutual agreement and shared decisions with the treating physician about recommended preventive treatment for their child’s asthma.

This study used a validated, reliable psychometric tool to determine parents’ beliefs and adherence to ICS use. To the best of our knowledge this is a rare study that compared parents’ beliefs about medicine in general, and about ICS that their child was using as a therapy for asthma. All children were treated only with ICS as long-term therapy, unlike another research group that was using all other asthma therapies [14,49].

## 5. Conclusions

Given the limitations of this study, which are the use of a questionnaire, not an objective method of measuring adherence, and a convenience sample of patients from one private specialist pulmonology practice, we can conclude that parents who think that medicines, in general, are not overused and not harmful have higher adherence. Also, parents with higher adherence are less concerned about ICS usage in therapy for their children’s asthma.

## Figures and Tables

**Table 1:** Pediatric patients with asthma descriptive characteristics (n = 148).

	Descriptive Statistics (N/%)
Age	
<5	61 (41.2%)
≥5	87 (58.8%)
Gender	
Male	99 (66.9%)
Female	49 (33.1%)
Allergic sensitization to aeroallergens	
Yes	77 (52%)
No	59 (39.9%)
Allergic sensitization to food allergens	
Yes	25 (16.9%)
No	101 (68.2%)
Pets (dog, cat, bird, hamster)	
Yes	49 (33.1%)
No	99 (66.9%)
Family history of atopy	
Yes	136 (91.9%)
No	12 (8.1%)
Comorbidities	
Yes	112 (75.7%)
No	36 (24.3%)
Hospitalization due to asthma ever	
Yes	30 (20.3%)
No	118 (79.7%)
Sports	
Yes	54 (36.5%)
No	80 (54.1%)
Hospitalization lasting 4 weeks	
Yes	3 (2%)
No	144 (97.3%)
ER visits for asthma ever	
Yes	58 (39.2%)
No	90 (60.8%)
Alternative treatments	
Yes	112 (75.7%)
No	36 (24.3%)
Asthma symptom control	
Well-controlled	57 (38.5%)
Partly controlled	47 (31.8%)
Uncontrolled	44 (29.7%)

N—number, %—percentage, ICS—inhaled corticosteroids, ER—emergency room.

**Table 2:** Descriptive characteristics of the parents of pediatric patients with asthma (n = 148).

	All Parents (n = 148)
Age	38.03 ± 6.22
Education level	
Primary school	0
High school	64 (43.2%)
Bachelor’s degree	26 (17.6%)
Master’s degree + PhD	58 (39.2%)
Employment status	
Unemployed	32 (21.6%)
Employed	115 (77.7%)
Pensioner	1 (0.7%)
Health condition	4 (IQR: 1)
Number of children	2 (IQR: 1)
Medical Education	
Yes	22 (14.9%)
No	126 (85.1%)
Smoker	
Yes	41 (27.7%)
No	107 (72.3%)
History of asthma?	
Yes	22 (14.9%)
No	126 (85.1%)
History of AD?	
Yes	23 (15.5%)
No	125 (84.5%)
History of AR?	
Yes	45 (30.4%)
No	103 (69.6%)
ICS treatment	
Yes	40 (27%)
Never	108 (73%)
TCS treatment	
Yes	63 (42.6%)
Never	85 (57.4%)
OCS treatment	
Yes	37 (25%)
Never	111 (75%)

AD—atopic dermatitis, AR—allergic rhinitis, ICS—inhaled corticosteroids, TCS—topical corticosteroids, OCS—oral corticosteroids.

**Table 3:** Beliefs about medicines general (BMQ-G).

	Items	Strongly Agree	Agree	Uncertain	Disagree	Strongly Disagree
Overuse	Doctors use too many medicines.	4 (2.7%)	19 (12.8%)	39 (26.4%)	74 (50.0%)	12 (8.1%)
	Doctors place too much trust in medicines.	8 (5.4%)	28 (18.9%)	62 (41.9%)	40 (27.0%)	10 (6.8%)
	If doctors had more time with patients they would prescribe fewer medicines.	15 (10.1%)	27 (18.2%)	57 (38.5%)	43 (29.1%)	6 (4.1%)
Harm	People who take medicines should stop their treatment for a while now and again.	11 (7.4%)	30 (20.3%)	60 (40.5%)	34 (23.0%)	13 (8.8%)
	Most medicines are addictive.	4 (2.7%)	18 (12.2%)	53 (35.8%)	57 (38.5%)	16 (10.8%)
	Most medicines are poisons.	1 (0.7%)	9 (6.1%)	43 (29.1%)	71 (48.0%)	24 (16.2%)
	Medicines do more harm than good.	2 (1.4%)	8 (5.4%)	28 (18.9%)	80 (54.1%)	30 (20.3%)
	Natural remedies are safer than medicines.	10 (6.8%)	24 (16.2%)	70 (47.3%)	35 (23.6%)	9 (6.1%)
Benefit	Medicines help many people to live better lives.	59 (39.9%)	76 (51.4%)	8 (5.4%)	0 (0%)	5 (3.4%)
	In most cases, the benefits of medicines outweigh the risks.	20 (13.5%)	64 (43.2%)	51 (34.5%)	12 (8.1%)	1 (0.7%)
	In the future medicines will be developed to cure most diseases.	24 (16.2%)	79 (53.4%)	37 (25.0%)	4 (2.7%)	4 (2.7%)
	Medicines help many people to live longer.	54 (36.5%)	67 (45.3%)	20 (13.5%)	4 (2.7%)	3 (2.0%)

Data are presented as whole numbers and percentages.

**Table 4:** Beliefs about medicines specific (BMQ-S).

	Items	Strongly Agree	Agree	Uncertain	Disagree	Strongly Disagree
Necessity	My child’s health, at present, depends on these medicines.	36 (24.3%)	79 (53.4%)	21 (14.2%)	10 (6.8%)	2 (1.4%)
	My child’s life would be impossible without these medicines.	13 (8.8%)	44 (29.7%)	47 (31.8%)	34 (23.0%)	10 (6.8%)
	Without these medicines, my child would be very ill.	16 (10.8%)	60 (40.5%)	50 (33.8%)	19 (12.8%)	3 (2.0%)
	My child’s health in the future will depend on these medicines.	8 (5.4%)	26 (17.6%)	71 (48.0%)	36 (24.3%)	7 (4.7%)
	These medicines protect my child from becoming worse.	54 (36.5%)	74 (50.0%)	13 (8.8%)	3 (2.0%)	4 (2.7%)
Concern	The fact that my child has to take medicines worries me.	26 (17.6%)	55 (37.2%)	15 (10.1%)	45 (30.4%)	7 (4.7%)
	I sometimes worry about the long-term effects of these medicines on my child.	36 (24.3%)	61 (41.2%)	25 (16.9%)	22 (14.9%)	4 (2.7%)
	My child’s medicines are a mystery to me.	4 (2.7%)	14 (9.5%)	19 (12.8%)	77 (52.0%)	34 (23.0%)
	These medicines disrupt my child’s life.	2 (1.4%)	5 (3.4%)	13 (8.8%)	90 (60.8%)	38 (25.7%)
	I sometimes worry about my child becoming too dependent on these medicines.	9 (6.1%)	30 (20.3%)	27 (18.2%)	59 (39.9%)	23 (15.5%)
	These medicines give my child unpleasant side effects.	1 (0.7%)	4 (2.7%)	29 (19.6%)	71 (48.0%)	43 (29.1%)

Data are presented as whole numbers and percentages.

**Table 5:** Medication Adherence Report Scale (MARS).

Items	Always	Often	Sometimes	Rarely	Never
I forget to give them	1 (0.7%)	2 (1.4%)	13 (8.8%)	67 (45.3%)	65 (43.9%)
I alter the dose	0 (0%)	1 (0.7%)	10 (6.8%)	21 (14.2%)	116 (78.4%)
I stop giving them for a while	3 (2%)	6 (4.1%)	23 (15.5%)	22 (14.9%)	94 (63.5%)
I decided to miss out on a dose	0 (0%)	1 (0.7%)	8 (5.4%)	20 (13.5%)	119 (80.4%)
I give less than instructed	0 (0%)	1 (0.7%)	6 (4.1%)	19 (12.8%)	122 (82.4%)
I do not give it because my child refuses it	0 (0%)	0 (0%)	3 (2%)	16 (10.8%)	129 (87.2%)

Data are presented as whole numbers and percentages.

## Data Availability

Data are contained within the article.
